# Equilibrative Nucleoside Transporter 2: Properties and Physiological Roles

**DOI:** 10.1155/2020/5197626

**Published:** 2020-12-03

**Authors:** Safaa M. Naes, Sharaniza Ab-Rahim, Musalmah Mazlan, Amirah Abdul Rahman

**Affiliations:** ^1^Department of Biochemistry & Molecular Medicine, Faculty of Medicine, Universiti Teknologi MARA, Cawangan Selangor, Kampus Sungai Buloh, 47000 Sungai Buloh, Selangor, Malaysia; ^2^Institute of Medical & Molecular Biotechnology, Faculty of Medicine, Universiti Teknologi MARA, Cawangan Selangor, Kampus Sungai Buloh, 47000 Sungai Buloh, Selangor, Malaysia

## Abstract

Equilibrative nucleoside transporter 2 (ENT2) is a bidirectional transporter embedded in the biological membrane and is ubiquitously found in most tissue and cell types. ENT2 mediates the uptake of purine and pyrimidine nucleosides and nucleobase besides transporting a variety of nucleoside-derived drugs, mostly in anticancer therapy. Since high expression of ENT2 has been correlated with advanced stages of different types of cancers, consequently, this has gained significant interest in the role of ENT2 as a potential therapeutic target. Furthermore, ENT2 plays critical roles in signaling pathway and cell cycle progression. Therefore, elucidating the physiological roles of ENT2 and its properties may contribute to a better understanding of ENT2 roles beyond their transportation mechanism. This review is aimed at highlighting the main roles of ENT2 and at providing a brief update on the recent research.

## 1. Introduction

The analysis of the human genome sequences indicates that approximately 4% of all genes encode transporter proteins [1]. The two membrane transporter protein superfamilies are the Solute Carrier (SLC) and ATP-Binding Cassette (ABC). More than four hundred transporters are organized into fifty-five families belonging to the SLC superfamily [2]. Thus, many of the important groups of transmembrane proteins are comprised of these transporters which are involved in the uptake of many different endogenous substrates, such as metabolites, and xenobiotic substrates, such as nutrients, drugs, and toxins. Structurally, the families that mediate nucleoside transport (NTs) are classified into two separate families of proteins: Na^+^-dependent concentrative transporters (CNTs; SLC28) and equilibrative bidirectional transporters (ENTs; SLC29) [3].

ENT family genes contain four members (ENT1, SLC29A1; ENT2, SLC29A2; ENT3, SLC29A3; and ENT4, SLC29A4). ENT family, except ENT4, transports the natural nucleosides across biological membranes by facilitating diffusion with broad substrate selectivity but comparatively lower affinity than CNTs [4]. ENT1-3 proteins transport both purine and pyrimidine nucleosides regardless of differences in substrate specificity [4]. ENT4, known as a pH-dependent transporter, can translocate adenosine at low pH [4, 5]. Previously, the classification of ENTs was made based on their sensitivity to inhibition by nitrobenzylmercaptopurine riboside (NBMPR) to equilibrative **s**ensitive (*es*; ENT1) or equilibrative insensitive (*ei*; ENT2). ENT2 is insensitive to the low concentrations (nM) of NBMPR, but it can be inhibited by the higher concentration (*μ*M) [6]. Thus, ENT2 was classified as a sodium-independent, NBMPR insensitive transporter that is involved in transporting the nucleosides and nucleobases of purine and pyrimidine molecules [4]. The ENT2 (SLC29A2) gene is located on chromosome 11q13.2. Human (h) ENT2 was initially encoded by human cDNAs which was isolated, independently, by two groups: the first group used a placental cDNA library while the second group used a HeLa cell cDNA library [6–8].

ENT2 protein is a glycosylated transport protein that contains 456 amino acid residues with a molecular mass of 50 kDa [9]. ENT2 amino acid sequence is 46% identical with ENT1. ENT2 and its family (ENT1, 3, and 4) are exclusively present in eukaryotes [4]. hENT2 and its orthologues in other mammalian species have a common structure and shared general functions with some differences [10] (Table 1). For example, hENT2 and rat (r) ENT2 have equal kinetic efficiencies for transport the purine and pyrimidine nucleosides and nucleobases [11].

Most of the common nucleoside transporter inhibitors such as NBMPR, dilazep, and dipyridamole are more specific for ENT1 rather than ENT2. However, 4-((4-(2-fluorophenyl) piperazin-1-yl) methyl)-6-imino-N-(naphthalen-2-yl)-1,3,5-triazin-2-amine (FPMINT) was found to be more selective towards ENT2 than ENT1 [12]. Recently, FPMINT derivative, a modified structure of FPMINT, was also more selective to ENT2 than ENT1 when compared to its parent compound FPMINT [13]. hENT2 (and intercellular hENT3) is the first identified mammalian nucleobase transporter protein in the human cells and tissues [10, 11].

The distribution of hENT2 is ubiquitous [10, 14]; however, its expression level is lower compared to hENT1 among cell types and tissues. Usually, ENT2 was reported to be localized at the basolateral part of polarized epithelial cells [15]. In contrast, ENT2 was also observed to be expressed at the apical part in the enterocytes and colon epithelial cells [16–18].

The exact function of ENT2 is still unclear, but because of its high capacity and low affinity in transporting a broad range of purine and pyrimidine nucleobases, e.g., hypoxanthine, cytosine, adenine, uracil, guanine, and thymine with high affinity for inosine [9, 19], ENT2 was presumed to play an essential role in maintaining nucleoside homeostasis [20]. Due to, moreover, ENT2 is suggested to play a key role in regulating the de novo nucleotide biosynthetic pathway by allowing the transportation of hypoxanthine when cells or tissues such as brain, bone marrow muscle, leukocytes, and erythrocyte cells are deprived of hypoxanthine [21, 22]. Hypoxanthine also serves as the source of purines in salvage pathway for nucleotide synthesis [22].

Most studies on the expression, localization, functions, and transport mechanism of ENTs have focused on ENT1. Studies on ENT2 remain at its infancy and merit further research. Table 1 describes briefly the current available information on the properties of ENT2 in different species.

## 2. ENT2 Splice Variants and Structure

In contrast to other transporter families, ENT genes appear to have a very low frequency of genetic variations [22, 23]. Therefore, it was suggested that the absence or mutations of ENT1 and ENT2 in embryos are lethal because, until now, there is no known human disease or syndrome related to mutations in both of them [24]. This might reflect the importance of these transporters.

Recently, several splice variants of the mammalian ENTs were identified in various tissues and subcellular localizations [25]. There are two splice variants that have been detected in hENT2 coding region [23, 26]. The splice variants in hENT2 produce both 36 kDa nucleolar protein designated HNP36 and another 32 kDa protein designated ENT2A [23, 27]. hHNP36 has been identified as the product of a delayed early response gene (DER12) [15, 22, 28]. A frameshift mutation resulted in mRNA of the splice variant to lack part of exon 4 and hence encoded a 326-residue truncated protein. The resultant protein lacks the first three transmembrane (TM) helices of hENT2 [6, 15, 22, 23, 26]. hENT2A is the second splice variant that is characterized by deletion of a 40 bp in exon 9. This yields a 301-residue C-terminally truncated protein [15, 22, 29]. Both variants are nonfunctional as nucleoside transporters and that might be because they do not possess the necessary domains for nucleoside transport [6, 15, 22, 23, 26, 27]. However, it was reported that nuclear hENT2 splice variants have a role in regulating cell proliferation [30]. Indeed, it has been shown that hENT2 splice variants can regulate the quantity and function of wild-type hENT2 at the nuclear envelope by nucleoside translocation into the nucleus for incorporation into DNA during the replication [30, 31].

**Figure 1:**
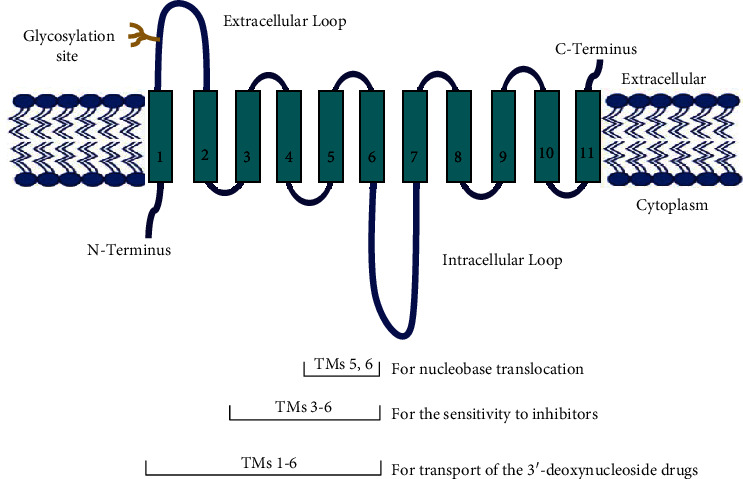
Topographical model of hENT2.

Currently, there is no data on the actual crystal structure or atomic resolution structure for any of the ENT family members [32]. The available ENT structural models are created by computational approaches based on crystal structures of major facilitator superfamily (MFS) transporters [26, 33, 34]. Based on this data, all ENTs are shown to share a common membrane topology [35] which consists of 11 transmembrane (TM) domains which are connected by short hydrophilic regions. However, there are larger loops linking TMs 1 and 2, made up of 27–41 residues and loops linking TMs 6 and 7, which are predicted to contain 66–80 residues [36]. These TMs have an intracellular/cytoplasmic (N-) amino terminus and extracellular carboxyl terminus (C-) [15] which was confirmed by glycosylation scanning mutagenesis and through the use of antipeptide antibodies as topological probes [6, 26, 35]. It was reported that ENT2 has two potential sites of N-glycosylation at Asn48 and Asn57 [37] in the large extracellular loop between TMs 1 and 2 [8]. The efficient targeting of hENT2 to the plasma membrane may occur via N-glycosylation; however, it will not affect the nucleoside transport kinetics [22, 25, 38] (Figure 1).

The important functional information about the inhibitor binding sites in TMs 3–6 and putative translocation pore have been revealed by chimeric studies with different hENT and rENT proteins [20, 26]. It was determined that the TM domains 1–6 of ENT2 are required for 3′-deoxynucleoside drugs transportation [7]. In addition, it was demonstrated that TM domains 5 and 6 in rENT2 are involved in nucleobase translocation [7], particularly to transport hypoxanthine [11]. Hence, the highly protected residues of the ENT family may have overlapping functional duties, which may be vital in explaining the ENT function.

## 3. ENT2 Localization and Expression

ENT2 was mainly reported to be localized at the basolateral side of polarized epithelial cells [14, 15, 29, 39]. It was demonstrated that ENT2 green fluorescence protein (GFP) fusions were targeted to the basolateral membrane when expressed in polarized renal epithelial cell line (MDCK) [15, 29]. On the other hand, there is evidence that ENT1 and ENT2 are also expressed at the apical membrane of the renal epithelium [18, 29, 40]. Furthermore, mRNA hENT2 and its protein were expressed at hepatocyte cell cytoplasm and borders [41]. It was documented that the staining for ENT2 protein in the epithelium and subepithelial cells of the colorectal biopsy showed uniform nuclear staining of all epithelial cells [42]. In addition, the studies that used differential biotinylation of the basolateral or the apical surface localized the majority of ENT2 to the apical surface of intestinal epithelia [16]. It was shown that adenosine and hypoxanthine uptake in Caco-2 polar enterocyte cells appeared to be relied mainly ENT2 apical expression [16–18, 43]; however, based on the immunocytochemical staining findings, the apical expression of ENT2 was demonstrated to be lower than the basolateral expression of ENT2 [18]. ENT2 was also found to be located on the apical membrane of Sertoli cells of the testis [44]. It was proposed that the expression of hENT proteins on the basolateral and apical membranes facilitate and allow the nucleosides and nucleoside drugs to transport from the intestinal lumen to the blood on the basis of their functional activity [18, 41].

ENT2 is expressed mostly at cell surfaces [22]. In general, hENT2 is distributed ubiquitously but differs in abundance among tissues and cell types. ENT2 mRNA is reported to be expressed in various tissues, including the heart, brain, salivary gland, thyroid gland, esophagus, thymus, stomach, vascular endothelium, pancreas, placenta, ovary, kidney, prostate, small intestine, and colon, with particularly high expression in skeletal muscle [6–9, 15, 17, 42, 45–47]. Furthermore, it was shown that mRNA level of ENT2 is highly expressed in the digestive system [2]. Based on the observation of in situ hybridization and immunohistochemical results, it was reported that mRNA hENT2 and its protein were present mainly in crypt cells of the duodenum; in the distal tubules, glomeruli, endothelial cells, and vascular smooth muscle cells of the kidney; in hepatocytes; and in chorionic villi sections (syncytiotrophoblast layer) of human placenta [41].

Besides that, the expression profile of hENT2 has been evaluated in several human normal and nonnormal tissue organs. Several studies have shown that the ENT2 expression is upregulated in nonhealthy tissues than in healthy tissues [2, 48]. For example, the mRNA expression level for ENT2 was significantly higher in inflamed colon than noninflamed tissues of inflammatory bowel disease patients [48]. The levels of ENT2 were observed to be 2-5.5-fold higher in breast, kidney, and prostate cancer cells than in normal cells [12]. The highest expression of ENT2 is in cancers derived from digestive organ tissues because of mRNA level of ENT2 is highly expressed in the digestive system [2]. Furthermore, a high level of the hENT2 expression was correlated with advanced stages of several cancers including hepatocellular carcinoma, mantle cell lymphoma, and ovarian carcinoma [49]. hENT2 gene and protein are also expressed in primary chronic lymphocytic leukemia (CLL) cells [14, 50]. For *in vitro* studies, the hENT2 gene expression in different cancer cell lines had been evaluated, and it was found to be highly expressed in several colorectal and rectal cell lines. hENT2 was detected in all sixteen colon cancer cell lines, and it was strongly expressed in four metastatic cell lines (Colo205, LoVo, SK-CO-1, and T84) and four primary cell lines (Caco-2, Colo320, HCT116, and HT29) [1]. Moreover, it was reported that the ENT2 gene expression was found to be highly expressed in four rectal adenocarcinoma cell lines (SW1463, SW837, HRA-16, HRT-18) [42].

## 4. Physiological Roles of ENT2

Despite the fact that the exact role of ENT2 is still not clear, the potential unique function for ENT2 is hypoxanthine transportation. The substrate supplies for nucleotide synthesis and maintaining the pool of intracellular nucleotides in case of reduced cellular nucleoside availability are provided by hypoxanthine. As a result, any interruption of ENT2 activity and/or expression could be detrimental to the cells by promoting the intracellular hypoxanthine conversion to xanthine and uric acid and then producing reactive oxygen species (ROS) as a by-product [12]. In addition, during muscle exercise and recovery, tissues such as skeletal muscle may take up adenosine and its metabolite inosine and hypoxanthine via ENT2 [6, 9, 51]. Furthermore, ENT2 and its family have an essential role in delivering nucleoside analogues to intracellular targets during the therapeutic strategies of viral infections and a lot of hematological and solid tumors [26, 52].

### 4.1. Drugs Transportation

ENT2 plays a crucial role in the disposition antiviral [15, 53] and anticancer [37] nucleoside analogues. These drugs work via incorporation into nucleic acids, or by interference with the metabolism of physiological nucleosides, or by interference with nucleic acid synthesis [54]. Some of the frequently used drugs in antiviral therapy and translocated by ENT2 (and another NTs) are zidovudine, zalcitabine, didanosine, and rivabirine [45]. The nucleoside derivatives commonly used in cancer treatment such as cytarabine [55], gemcitabine [55], fludarabine [50], cladribine [5], clofarabine [5], trifluridine [56], 5-fluorouracil [57], and 50-deoxy-5-fluorouridine (5′dFUR) [58] are translocated by a variety of NTs including ENT2 [4, 37]. For example, gemcitabine has activity in various solid tumors and some hematological malignant diseases [54]. Gemcitabine is transported by ENT2 with low affinity and high capacity compared to ENT1 [59]. It has been shown that the disruption of ENT2 localization in plasma membrane leads to a reduced uptake of gemcitabine in pancreatic cancer, consequently, increasing the chemoresistance to this drug [2]. Another example is 5-fluorouracil, a widely used chemotherapeutic drug for the treatment of gastrointestinal cancer especially for colorectal cancer [60] and metastatic breast cancers [57]. It was often associated with significant normal-tissue toxicities [57]. Low level of hENT1 was correlated with sensitive response to 5-fluorouracil; however, 5-fluorouracil cytotoxicity was detected when ENT inhibitor, NBMPR was used at 10 *μ*M NBMPR. Since hENT1 is sensitive to the inhibition of NBMPR while hENTs are not, this observation points to a possible involvement of hENT2 [60].

Trifluridine, a chemotherapeutic drug for metastatic colorectal cancer [61], is a substrate for ENT1 and ENT2. Normally, the efflux of trifluridine from the inside of enterocytes is facilitated by ENT-mediated transport at the basolateral side. Consequently, the inhibition of ENT1 and ENT2 leads to an increase in the accumulation of trifluridine inside the human small intestinal epithelial cell (HIEC), resulting in cellular toxicity [62]. It has also been suggested that hENT2 may play a role in fludarabine responsiveness in chronic lymphocytic leukemia (CLL) patients [50].

Thus, the importance of ENT2 is not just because of its function in the provision of nucleosides and nucleobase, which are derived from the diet or produced by tissues by salvage pathways or *de novo* biosynthetic pathways [30] but also because of its ability to mediate the uptake and efflux of nucleosides and therapeutic analogues. However, disruption of expression of ENT2 may consequently affect the uptake or efflux of anticancer drugs in/out of the cancer cells, so it may lead to drug resistance during chemotherapy or may improve the strategies of cancer therapeutic possibilities. Accordingly, the process mediated by ENTs has been recognized by the therapeutic strategies as a potential target [10].

### 4.2. Adenosine Signaling

In cellular signaling by the endogenous purine nucleoside, adenosine, the ENT family plays a main role in the regulation process by affecting the available concentrations of adenosine to the G-protein-coupled membrane adenosine subtype receptors (A1, A2A, A2B, and A3) [63]. Increasing or decreasing the activity of adenosine receptors may cause different diseases, like hypertension, cancer, diabetes mellitus, insulin resistance, or obesity [64]. In this sense, the control mechanism of purinergic pathway is very important in the intestine especially during infection and inflammation, which could evolve to cancer [65]. During the purinergic regulation, most cells will release the secretion of nucleotide and the derivative nucleosides like adenosine from ATP in an organized process to provide the purinergic responses by primary components [66].

Adenosine can be transported into cells by CNTs and ENTs [67]. hCNT1 and hCNT2 are high-affinity inward transporters for adenosine. However, it was found that hCNT1 was not able to efficiently translocate the adenosine [17]. The apical location of hCNT2 made it a suitable candidate to modulate intestinal functions during inflammation; however, so far, there is no clear evidence that hCNT2 regulates the extracellular adenosine levels in intestinal cells [17]. On the contrary, ENTs, especially ENT2, seem to be involved in intestinal purinergic regulation. It was reported that under some of the pathophysiological conditions, there are changes in human intestinal transporter expression including NT proteins. In human duodenum, the selected NT genes are downregulated as an early response to high elevation. The mRNA levels of hCNT1 and hCNT2 are rapidly decreased while the mRNA amounts of hENT2 are barely modified [17, 68]. ENT1 was shown to be a major player in the regulation process of adenosine-mediated purinergic signaling in several organs and cell types, including brain [69], heart [70], human umbilical vein endothelial cells (HUVEC) [17], and liver [71]. In contrast, ENT2 was suggested to be the major player in regulation process of adenosine levels in the gastrointestinal tract [16, 17] particularly, in the colon [17].*In vitro* studies, it was shown that when the Caco-2 cells grown on transwell plates, it will have the ability to remove adenosine significantly from the culture medium to inside cells; then, it will be accumulated there [16, 17].

On the other hand, it was found that ENT2 knockout increased the levels of adenosine in bronchoalveolar fluid and alveolar space [26, 72, 73]. Accordingly, it was reported that the important regulator of adenosine levels in the alveolar space might to be ENT2 [72]. Recently, it was found that the hENT1 and hENT2 proteins in HUVECs have the possibility of intracellular pH regulating adenosine transport [49].

### 4.3. Regulation of Cell Cycle Progression

The specificity of different substrates and the action of ENT mode might support several and perhaps complementary roles in modifying nucleoside bioavailability which may be correlated with more particular functions and/or various regulatory roles for the different subtypes of ENT protein. Accordingly, it was proposed that ENTs might contribute to maintaining metabolic homeostasis [49]. During the salvage pathway, hENTs may have a critical role by maintaining the nucleoside and nucleotide pool regulation. Even so, the nucleotide and nucleoside pool regulation may also have regulatory relations. Based on cell requirements, the required amount of nucleosides/nucleotides will be delivered from cytoplasmic to nuclear pools [49, 74]. Furthermore, the integrity of DNA replication and processes of mutagenic that is related to carcinogenesis may be determined by irregularity in deoxynucleoside triphosphate (dNTP) pool [75, 76]. Recently, the nucleoside and nucleotide pool for effective DNA synthesis and cell cycle progression were suggested to be controlled by hENT2 and two novel nuclear smaller hENT2 isoforms [30, 49]. It was shown that nuclear hENT2 splice variants are widely expressed among proliferative cells (cancer cells) of different tissue types and cell lines and have high levels of mRNA of nuclear hENT2 isoforms compared to nonproliferative tissue types. Thus, it was suggested that the correlation between regulation of alternative splicing of hENT2 and proliferative status of the cell indicates that the nuclear hENT2 variants play a role in cell proliferation and cell cycle regulation [30]. These pieces of evidence indicate that the required rapid supply of nucleotides into the nucleus during DNA replication was supported by hENT2 and their isoforms.

## 5. ENT2 Is Associated with Cancer Progression

Due to the importance of nucleoside and nucleotide in many physiological processes such as DNA synthesis, the homeostasis of these molecules must be highly protected. The nucleoside influx mostly relied on ENT transporters [49]. Accordingly, some of the pathophysiological condition such as cancer, which requires abnormal high levels of nucleotides, the change in the expression, and/or activity of ENT transporters, may play a crucial role in cancer progression and therapeutic mechanisms.

The expression profile of hENT2 has been evaluated in several solid cancers, including gastrointestinal, breast, pancreatic, kidney, colorectal, and prostate cancers [12, 42, 60, 77, 78]. Indeed, the advanced stages in several cancers [49] including mantle cell lymphoma, hepatocellular carcinoma, and ovarian carcinoma have been correlated with the high level of ENT2 expression [49, 79, 80]. However, it was reported that a high level of the ENT2 expression in ovarian carcinoma was correlated (but not significantly) with progression-free survival or overall survival [79]. Furthermore, it was shown that adenosine significantly increased the expression of ENT2-4 which lead to an increase in the effective delivery of adenosine into pancreatic cancer cells; consequently, it was suggested that ENTs may contribute to the adenosine effect on the suppression of pancreatic cancer effectively [81]. Thus, there remains a considerable amount of work to be done before the clinical significance of ENT2 nucleoside-analogues is completely understood in tumor tissues.

## 6. Conclusions

The clinical importance of ENT2 is becoming more recognized; however, the role of hENT2 as a biomarker in the management of the different cancer types requires further research to specify whether ENT2 gene may play a role in inducing the biological process to suppress the cancer cells and/or improve the possibilities of cancer therapeutic strategies.

## Figures and Tables

**Table 1:** Properties of mammalian ENT2 proteins.

ENT2 transporter	Gene location	Amino acid	Tissue distribution	Permeant selectivity
hENT2	11q13.2	456	Ubiquitous	Purine and pyrimidine nucleoside and nucleobases
mENT2	19; 19 A	456	Brain, NG108-15 cells [7]	Purine and pyrimidine nucleoside and adenine
rENT2	1q43	456	Intestine, brain, epididymis [7]	Purine and pyrimidine nucleoside and nucleobases except cytosine
rbENT2	Un	456	nd	Purine and pyrimidine nucleoside and hypoxanthine

Mammalian ENT2 proteins differ in the distributions and their abilities to transport some nucleobases. h: human; m: mouse; r: rat; rb: rabbit; Un: unplaced; nd: not determined.

## Supplementary Materials

Supplementary Materials Supplementary Document 1: graphical abstract.
