# Supplementary material for: Equilibrative Nucleoside Transporter 2: Properties and Physiological Roles
Source: Biomed Res Int. 2020 Dec 3;2020:5197626. doi: 10.1155/2020/5197626 (PMC7732376; doi:10.1155/2020/5197626)
Supplement: Supplementary Materials — Supplementary Document 1: graphical abstract. [file 5197626.f1.pptx]

## Slide 1
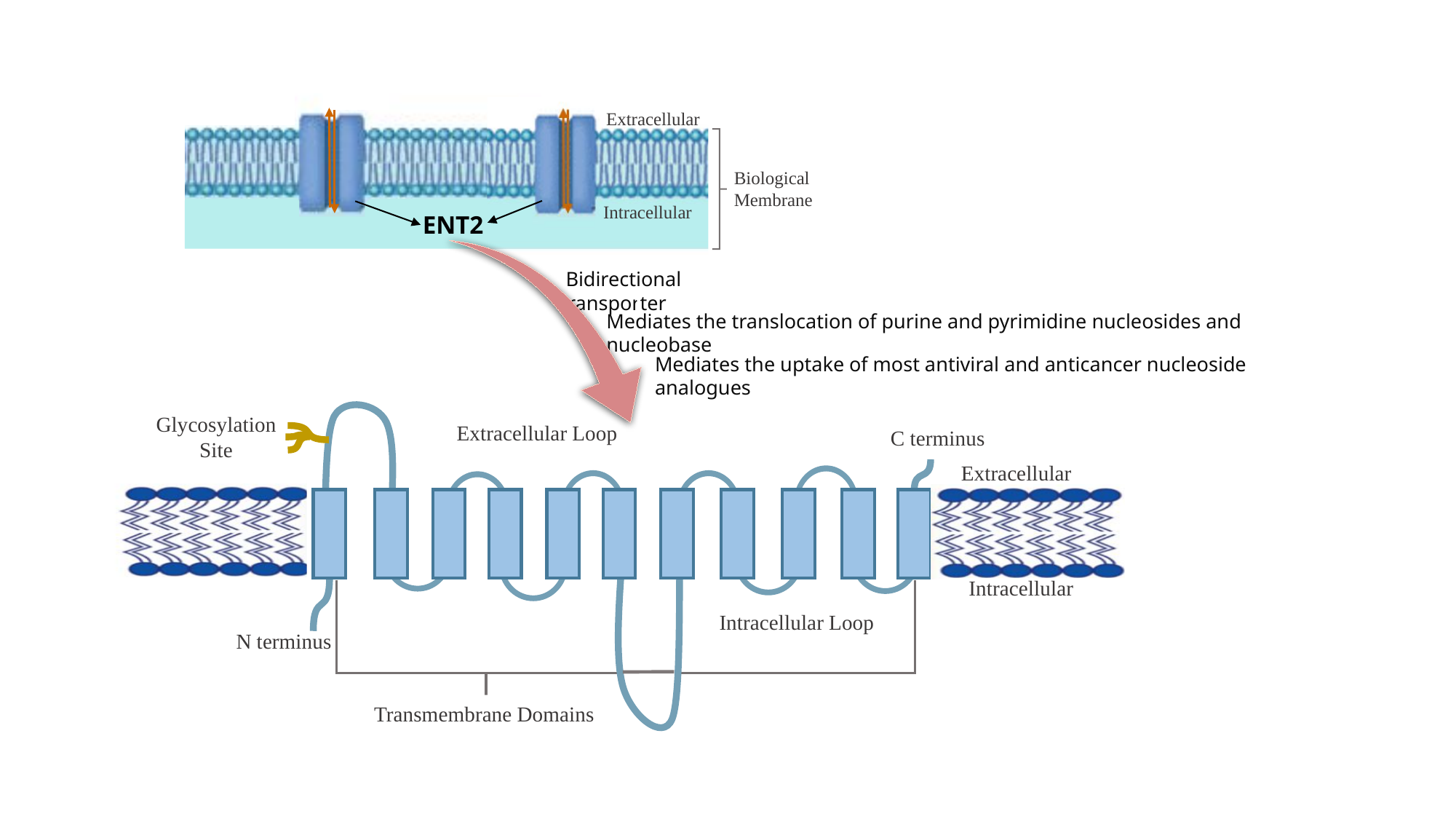

ENT2
Biological
Membrane
Extracellular
Intracellular
 Bidirectional transporter
Mediates the translocation of purine and pyrimidine nucleosides and nucleobase
Mediates the uptake of most antiviral and anticancer nucleoside analogues
Glycosylation Site
Extracellular Loop
C terminus
Extracellular
Intracellular Loop
N terminus
Transmembrane Domains
Intracellular
